# Use of the Osteoporosis Self-Assessment Tool as a Screening Tool for Osteoporosis in Saudi Postmenopausal Women

**DOI:** 10.7759/cureus.37755

**Published:** 2023-04-18

**Authors:** Fahad Alshahrani, Yazeed A Alsulaiman, Yasser M Almashari, Nawaf K Alawad, Saad A Almousa, Yazeed Allarakia, Bader A Aljaafri

**Affiliations:** 1 Family Medicine, King Abdulaziz Medical City Riyadh-Ministry of the National Guard Health Affairs, Riyadh, SAU; 2 Collage of Medicine, King Saud Bin Abdulaziz University for Health Sciences, Riyadh, SAU; 3 Family Medicine, King Abdullah International Medical Research Center, Riyadh, SAU; 4 College of Medicine, King Saud Bin Abdulaziz University for Health Sciences, Riyadh, SAU

**Keywords:** postmenopausal women, osti, bmd, osteopenia, osteoporosis

## Abstract

Background: Osteoporosis is commonly referred to as the “silent disease,” as bone loss is gradual and asymptomatic. In older women and men, osteoporosis can lead to increased bone fragility, thus increasing the risk of fractures. These fractures are associated with healthcare costs, physical disabilities, impaired quality of life, and mortality. Therefore, the study’s main objective was to assess the applicability of the osteoporosis self-assessment tool (OST) in predicting osteoporosis in Saudi postmenopausal women who are 60 years of age and older and to give a thorough understanding of how such a method can aid in the early diagnosis of osteoporosis in Saudi Arabia and give physicians enough time to treat it.

Methods: This study was done at King Abdulaziz Medical City, Riyadh, Saudi Arabia, where we included postmenopausal Saudi women 60 years of age and older who took the bone mineral density (BMD) test in the family medicine department. The approximate target population in this group, between the years 2016 and 2022, was 2969 patients. All data was taken from the BestCare database at King Abdulaziz Medical City in Riyadh. Data were typed in an Excel sheet (Redmond, USA), then transferred to the R Studio software. The data collection method was chart review, so no informed consent was needed from patients. Names and medical record numbers were not stored.

Results: The study included 2969 participants. According to the bone mineral density (BMD) T score results, 490 participants (16.5%) were normal, 1746 participants (58.8%) had osteopenia, and 733 participants (24.7%) suffered from osteoporosis. BMD T scores for normal, osteopenia, and osteoporosis participants were -0.6 (-0.9, -.3), -1.8 (-2.1), and -3 (-3.5, -2.7), consecutively. Estimated OSTI scores for those patients were 2 (0, 4), 1 (-2, 3), and -1 (-4, 1), consecutively. According to the OSTI score for normal participants, 4.29% were classified as being at high risk of osteoporosis. A high risk of osteoporosis was identified in 0.74% of those with osteopenia. 27.83% of osteoporosis patients were classified as being at high risk of osteoporosis. To differentiate normal individuals from those with osteopenia, the cutoff value with optimal sensitivity was 3.5. At such a cutoff value, the test sensitivity was 81.04%. To differentiate normal participants from those with osteoporosis, the cutoff value with optimal sensitivity was 2.5. At such a cutoff value, the test sensitivity was 86.49%. To differentiate osteopenia from osteoporosis patients, the cutoff threshold with optimal sensitivity was 1.5. At such a threshold, sensitivity was 78.44%.

Conclusion: OSTA is a simple and validated tool that can identify subjects at increased risk of osteoporosis. Its use could facilitate a more cost-effective use of BMD; by avoiding measurements in low-risk groups.

## Introduction

Osteoporosis is commonly called the "silent disease" as bone loss is gradual and asymptomatic in older women and men, increasing bone fragility and thus raising the risk of fractures [1]. These fractures are associated with higher healthcare costs, physical disabilities, impaired quality of life, and a higher mortality rate [1,2]. It was also reported in a systematic review that osteoporosis-related fractures in the USA were linked with high total medical and hospitalization costs [3].

The prevalence of osteoporosis in Saudi Arabia is increasing [4]. Since osteoporotic fractures have become more prevalent with advancing age, preventing osteoporosis and its complications is a critical public health concern [1]. Early diagnosis and treatment of osteoporosis are crucial in preventing fractures in the elderly and high-risk individuals [5]. According to the World Health Organization (WHO), a bone mineral density (BMD) ≤ −2.5 standard deviations (SD) below the young adult mean (or a T-score ≤ −2.5) indicates osteoporosis, and a T-score value at any site between ≤−1.0 and >−2.5 indicates a low bone mass or osteopenia [6]. The gold standard in diagnosing osteoporosis is dual-energy X-ray absorption (DXA). However, availability is limited in many hospitals; only 15%-25% of those patients who suffer a fragility fracture are scanned and treated [7]. Other than the availability, the operating cost of a DXA and the need for a specialized technician limit its usage in hospitals [8]. Due to the lack of DXA, diagnosing and treating osteoporosis in Saudi Arabia is difficult [9].

The osteoporosis self-assessment tool (OST) has been validated in numerous populations across the globe and has demonstrated its ability to help identify those most at risk for osteoporosis or low BMD [10,11]. Implementing OST and other screening modalities can spare elders many future obstacles, such as limiting their quality of life, being dependent on others, and decreasing their life expectancy [12,13]. OST could identify the population at risk, but characteristics such as ethnicity, gender, and age could alter the outcomes [5,14]. Thus, these characteristics should be considered while using OST worldwide for better outcomes [5,14]. Along with that, the OST cutoff should be revamped based on these characteristics [5]. Assessing osteoporosis in such an increasing population will benefit them and remove a massive burden from the country's healthcare system since managing fractured osteoporotic patients can be expensive [15]. In an Irish study, after evaluating more than 36,000 patients, it was found that OST performed well and appeared robust across multiple sub-group analyses. It concluded that OST is a simple and effective tool to help identify patients with low BMD or osteoporosis [16]. Lin et al. also revealed in a study that OST is better than grip strength, gait speed, and calf circumference in predicting osteoporosis [17].

Nieves et al. found that men tend to have a higher percentage of lean body mass than women, and women also tend to lose bone mass faster [18]. A recent study found that, compared to men, postmenopausal women have a two-fold increase in osteopenia and a four-fold increase in osteoporosis rates [19]. OST is a crucial osteoporosis screening tool for identifying high-risk individuals and arranging for a DXA scan. Moreover, with early screening, the financial and overall burden of osteoporosis on the healthcare system is reduced by enabling early disease assessment and treatment. Therefore, this study's main objective is to assess the applicability of OST in predicting osteoporosis in Saudi postmenopausal women who are 60 years of age and older and to give a thorough understanding of how such a method can aid in the early diagnosis of osteoporosis in Saudi Arabia.

## Materials and methods

This study was done at King Abdulaziz Medical City, the Family Medicine Department, and the National Guard Health Affairs Hospitals (NGHA) in Riyadh, Saudi Arabia. 

The target population of the study was postmenopausal Saudi women 60 years of age and older who took the bone mineral density test between 2016 and 2022 in King Abdulaziz Medical City, Riyadh, Saudi Arabia. The approximate target population in this group is 2969 patients. 

Postmenopausal Saudi women who were 60 years of age or older were included in the study. The exclusion criteria included patients with a history or evidence of metabolic bone disease, hyper- or hypoparathyroidism, a history of Paget’s disease, a history of osteomalacia, a history of renal osteodystrophy or osteogenesis imperfecta, a history of the presence of cancer(s) with known metastasis to bone, a history of or evidence of significant renal impairment, a history of >1 ovary removed, a history of both hips previously fractured or replaced, or prior use of any osteoporosis medications bisphosphonate, denosumab or anabolic. 

The data collection method was a retrospective chart review; all data were taken from the BestCare database at King Abdulaziz Medical City in Riyadh. Data were typed in an Excel sheet (Redmond, USA), then transferred to the R Studio software. Variables included age, sex, height, weight, BMI, and BMD results at three sites (lumbar spine, total hip, neck of femur). 

The research was done through an equation called the osteoporosis self-assessment tool index using the following method: (OSTi) = [weight (kg) - age (years)] divided by 5, rounded to the nearest integer, and compared the AUC values, sensitivity, and specificity of OSTi for patients at each skeletal site (lumbar spine, femoral neck, and total hip), those with a T-score threshold of ≤ -2.5 at any sites. 

The data were collected, reviewed, and then fed to R Studio software (version 2022.02.3, The R Foundation for Statistical Computing, Boston, USA) for data analysis. The normality of the data distribution was checked using the Kolmogorov-Smirnov test, histogram, and Q-Q plots. Continuous variables were presented as medians (25th percentile, 74th percentile). They were compared using the Kruskal-Wallis rank sum test because they were not normally distributed. Categorical variables were presented as frequencies (%) and compared using Pearson's Chi-squared test. Bar charts and scatter plots were used to demonstrate the OSTI score for each study group and correlate it with the BMD T score. They were built using the “ggplot2” and “smplot2” R packages. 

Receiver operating characteristic curve (ROC) analysis was done to assess OSTI score performance, and the findings were plotted using the pROC R package. Sensitivity, specificity, accuracy, positive predictive value, negative predictive value, and area under the curve (AUC) were expressed for each level of classification (normal vs. osteopenia, osteopenia vs. osteoporosis, and normal vs. osteoporosis), and then an overall mean was estimated. p < 0.05 was considered statistically significant.

## Results

The study included 2969 participants. According to the bone mineral density T score results, 490 participants (16.5%) were normal, 1746 participants (58.8%) had osteopenia, and 733 participants (24.7%) suffered from osteoporosis. Table 1 summarizes the baseline as well as clinical characteristics of those patients. Patients with osteoporosis were significantly older (74 (66, 76) years) than those with osteopenia (70 (66, 76) years) and normal participants (68 (66, 73) years; P<0.001). They also had a significantly lower weight (66 (57, 77.5) kg) than those with osteopenia (74 (65.2, 83.38) kg) and those who were normal (79 (70, 90) kg; p<0.001). Normal participants were significantly taller (155 (150.1, 159) m) than those with osteopenia (153 (148.55, 156) m) and those who suffered from osteoporosis (151 (147, 155) m; p< 0.001). Overall, osteoporosis patients had a significantly lower BMI (29.52 (25.29, 33.69)) than osteopenia patients (31.76 (28.15, 35.67)) and normal participants (33.08 (29.62, 37.03)).

**Table 1:** participants’ baseline and clinical characteristics. 1. Median (IQR); n (%). 2. Kruskal-Wallis rank sum test; Pearson's Chi-squared test

Characteristic	Normal, N = 490^1^	Osteopenia, N = 1,746^1^	Osteoporosis, N = 733^1^	Overall, N = 2,969^1^	p-value^2^
Age (years)	68.00 (66.00, 73.00)	70.00 (66.00, 76.00)	74.00 (68.00, 81.00)	70.00 (66.00, 77.00)	<0.001
Weight (kg)	79.15 (70.00, 90.00)	74.00 (65.20, 83.38)	66.00 (57.00, 77.50)	73.00 (64.00, 83.00)	<0.001
Height (m)	155.00 (150.10, 159.00)	153.00 (148.55, 156.00)	151.00 (147.00, 155.00)	152.50 (148.00, 156.30)	<0.001
BMI	33.08 (29.62, 37.03)	31.76 (28.15, 35.67)	29.52 (25.29, 33.69)	31.39 (27.69, 35.57)	<0.001
BMD T score	-0.60 (-0.90, -0.30)	-1.80 (-2.10, -1.50)	-3.00 (-3.50, -2.70)	-1.90 (-2.50, -1.30)	<0.001
OSTI score	2.00 (0.00, 4.00)	1.00 (-2.00, 3.00)	-1.00 (-4.00, 1.00)	0.00 (-2.00, 3.00)	<0.001
OSTI risk					<0.001
Low	423.00 (86.33%)	1,304.00 (74.68%)	370.00 (50.48%)	2,097.00 (70.63%)	
moderate	46.00 (9.39%)	272.00 (15.58%)	159.00 (21.69%)	477.00 (16.07%)	
High	21.00 (4.29%)	170.00 (9.74%)	204.00 (27.83%)	395.00 (13.30%)	
Location					<0.001
lumbar spine	226.00 (46.12%)	950.00 (54.41%)	519.00 (70.80%)	1,695.00 (57.09%)	
femoral neck	235.00 (47.96%)	699.00 (40.03%)	158.00 (21.56%)	1,092.00 (36.78%)	
proximal femur	24.00 (4.90%)	90.00 (5.15%)	49.00 (6.68%)	163.00 (5.49%)	
distal radius	4.00 (0.82%)	7.00 (0.40%)	6.00 (0.82%)	17.00 (0.57%)	
forearm	1.00 (0.20%)	0.00 (0.00%)	1.00 (0.14%)	2.00 (0.07%)	

**Figure 1:**
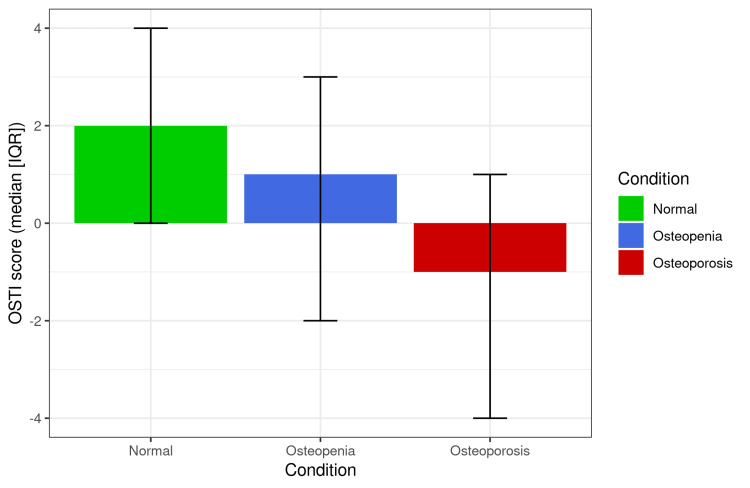
Bar chart demonstrating OSTI score (median [IQR]) according to participants’ actual condition

**Figure 2:**
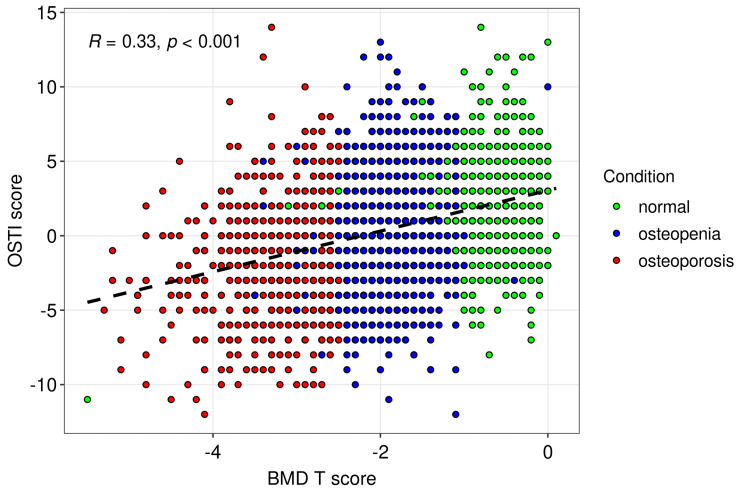
Demonstrates the correlation between OSTI and BMD T scores. The strength of the correlation was moderate (correlation coefficient = 0.33) and highly statistically significant (p<0.001).

BMD T scores for normal, osteopenia, and osteoporosis participants were -0.6 (-0.9, -.3), -1.8 (-2.1,-1.5), and -3 (-3.5, -2.7), consecutively (p<0.001). Estimated OSTI scores for those patients were 2 (0, 4), 1 (-2, 3), and -1 (-4, 1), consecutively (Figure 1; p< 0.001). According to the OSTI score for normal participants, 86.33% of individuals were classified to be at low risk of osteoporosis, 9.39% were classified as being at moderate risk of osteoporosis, and 4.29% were classified as being at high risk of osteoporosis. 74.68% of osteopenia participants were classified as being at low risk, 15.58% were classified as being at moderate risk, and 0.74% were classified as being at high risk of osteoporosis. 50.48% of osteoporosis patients were classified as being at low risk, 21.69% as being at moderate risk, and 27.83% as being at high risk of osteoporosis (p<0.001). Most of the participants had a bone scan on their lumbar spine (57.09%) or femoral neck (36.78%) (Figure 2).

We used receiver operator characteristic curve (ROC) analysis to evaluate the ability of the proposed OSTI score to differentiate normal from osteopenia participants, osteopenia from osteoporosis participants, and normal from osteoporosis participants. Table 2 summarizes the OSTI score performance. To differentiate normal individuals from those with osteopenia, the cutoff value with optimal sensitivity was 3.5. At such a cutoff value, the test sensitivity was 81.04%, the specificity was 32.45%, the accuracy was 70.39%, the positive predictive value was 81.04%, and the negative predictive value was 32.45%. To differentiate normal participants from those with osteoporosis, the cutoff value with optimal sensitivity was 2.5. At such a cutoff value, the test sensitivity was 86.49%, the specificity was 46.12%, the accuracy was 70.32%, the positive predictive value was 70.6%, and the negative predictive value was 69.54%. To differentiate osteopenia from osteoporosis patients, the cutoff threshold with optimal sensitivity was 1.5. At such a threshold, sensitivity was 78.44%, specificity was 39.81%, accuracy was 51.23%, positive predictive value was 35.36%, and negative predictive value was 81.48%.

**Table 2:** Performance of OSTI score at selected cutoff values. * AUC: Area under the curve.

	Normal vs. osteopenia	Normal vs. osteoporosis	Osteopenia vs. osteoporosis	Overall
Optimal cutoff value	3.5	2.5	1.5	-
Sensitivity	81.04%	86.49%	78.44%	81.99%
Specificity	32.45%	46.12%	39.81%	39.46%
Accuracy	70.39%	70.32%	51.23%	63.98%
Positive predictive value	81.04%	70.6%	35.36%	62.33%
Negative predictive value	32.45%	69.54%	81.48%	61.16%
AUC	62.6%	66.6%	76.6%	68.6%

**Figure 3:**
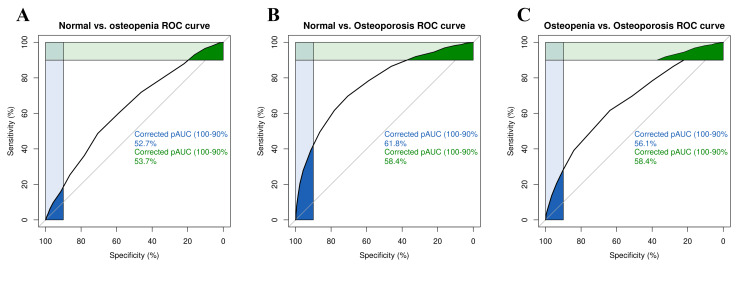
ROC plots with related AUC (area under the curve): normal vs. osteopenia; normal vs. osteoporosis; osteopenia vs. osteoporosis.

Figure 3 shows ROC curves for OSTI scores at different levels of classification with related area under the curve (AUC). OSTI achieved an AUC of 62.6% for differentiating normal from osteopenia participants, an AUC of 66.6% for differentiating osteopenia from osteoporosis patients, and an AUC of 76.6% for differentiating normal from osteoporosis participants. The overall AUC was 68.6%.

## Discussion

Preventing osteoporosis must be a goal for healthcare providers and the public. Preventing osteoporosis needs good screening programs and also community awareness. People must know more about the disease and how to screen for and prevent it. Studies have been conducted on awareness and self-efficacy, which show the importance of education in preventing such a disease. Moreover, educational programs are shown to be the most effective way of osteoporosis prevention. Furthermore, the population's knowledge, health beliefs, and self-efficacy must be assessed before implementing any educational program [20-23]. Based on an analysis of 860 postmenopausal women from various Asian regions, the Osteoporosis Self-Assessment Screening Tool for Asians (OSTA) was established in 2001 [24]. OSTA is a convenient and valid screening tool for predicting the risk of osteoporosis in postmenopausal women, despite only considering age and weight [25]. Diverse populations and diagnostic criteria may affect OSTA efficacy and cut-off value [26]. The tool must be revalidated according to the local demographic profile to apply OSTA clinically for osteoporosis screening [27]. 

We have 733 patients diagnosed with osteoporosis based on the T-score of the femoral neck, lumbar spine, proximal femur, distal radius, or forearm. Most participants had a bone scan on their lumbar spine (57.09%) or femoral neck (36.78%). Researchers have suggested that the reference standard should be based on BMD measurements of the femoral neck and total hip [28,29]. Diagnosing osteoporosis using multiple BMD measurements (lumbar spine, total hip, or femoral neck) is possible. Still, the IOF Epidemiology and Quality of Life Working Group recommends that studies of the descriptive epidemiology of osteoporosis include measurements of the femoral neck [30]. However, the number of patients would have been underestimated if the T-score had been measured only in the femoral neck (n = 158). Moreover, osteoporosis diagnosed at any site could predict future fractures at other sites [11]. A T-score ≤ −2.5 at any site other than just on the femoral neck can identify osteoporosis [26]. 

We found out that osteoporosis patients had a significantly lower BMI (29.52 [25.29, 33.69]) than osteopenia patients (31.76 [28.15, 35.67]) and normal participants (33.08 [29.62, 37.03]). On the contrary, another study showed that 71% of patients diagnosed with osteoporosis had a normal weight (BMI, 18.5-24.9), 3% were underweight (BMI <18.5), 21% were overweight (BMI, 25-29.9), and 5% were obese (BMI >30) [31]. Moreover, only one (0.2%) was underweight and also had osteoporosis (100%) [31]. The Cochran-Armitage Trend Test suggests the risk of osteoporosis increases as BMI decreases, P<0.0001 [31]. Other studies have shown that a lower BMI is associated with osteoporosis, like our results [32-34]. We also found out that patients with osteoporosis were significantly older than those with osteopenia and normal participants. Also, Jiang X. et al. and Yang Y. et al. found that age increases the risk of osteoporosis and fractures for older postmenopausal women [26,32]. Another study showed weight association was more vital than age [35].

BMD T scores for osteoporosis patients were -3 (-3.5, -2.7). The estimated osteoporosis self-assessment tool index (OSTI) scores for those patients were -1 (-4, 1), while in another study by Yang Y. et al., the OSTI varied from -10 to 8 [26]. According to the OSTI score, 50.48% of osteoporosis patients were classified as low-risk and 27.83% as high-risk for osteoporosis. Yang Y. et al. found that, based on the osteoporosis risk categories used in Asian women [24], 59.45% of the women had a low risk, and 40.55% had an increased risk of osteoporosis [26]. Another study by Kung AW et al. showed that 55% had an increased risk of osteoporosis, while the remaining 45% were in the low-risk category [36]. Also, Nguyen TV et al. showed in their study that 40% of women had a T score greater than -2.5 [35]. 

The correlation between OSTI and BMD T scores was moderate (correlation coefficient = 0.33). The same results in a study by Yang Y. et al. showed a moderately positive correlation between OSTA index values and BMD T-scores at different sites [26]. Adjustment of the original cut-off values, determined by the results of postmenopausal elderly women, is often needed to warrant optimal performance as the sensitivity and specificity may differ depending on gender, age, and ethnicity. Therefore, comparisons to other studies with different cut-off values are essential to initiate a fortified base of information about their utility that allows primary care physicians to generalize their results.

Compared to our paper, a study that was performed on 257 men of Indian descent aged 50 years or older evaluated the sensitivity, specificity, positive predictive value, and negative predictive value for various cut-offs of the OST index and found that on said population, a cut-off of two or less had a sensitivity of 95.7% and a specificity of 34%. The positive predictive value, together with the negative predictive value, was 24.4% and 97.7%, respectively [11]. Another study performed on 202 Portuguese men utilized a cut-off of less than three, in which the sensitivity was 73.5%, specificity was 58.3%, positive predictive value was 26.3%, and negative predictive value was 91.6% [37]. With regards to the performance of it in different populations of women, a study that was performed on healthy women (age range: 40-96 years) from a hospital in Chengdu region, China, found that with a cut-off of < -1 (LS T-score < −1) the sensitivity equals 56.9% and specificity is close to 87.7% [14]. The results of the receiver operating characteristic (ROC) curves that were constructed at different levels of classification can be contrasted to a paper that was performed on postmenopausal Chinese women, in which the results revealed that the area under the ROC curve for the OSTA index for the femoral neck, total hip, and L1-4 lumbar spine were 0.824, 0.824, and 0.776, respectively [26]. 

Limitations

The study was conducted on a relatively small sample size of 733 patients diagnosed with osteoporosis based on the T-score of the femoral neck, lumbar spine, proximal femur, distal radius, or forearm. A larger sample size would increase the generalizability of the findings. The study was conducted at a single center, which limits the generalizability of the findings to other populations and healthcare settings. The study only assessed BMI and age as potential risk factors for osteoporosis, which limits the scope of the study's findings. Other important risk factors, such as family history, smoking, and physical activity, were not evaluated. Lack of information on confounding factors: the study did not collect information on potential confounding factors that may impact bone health, such as dietary habits, physical activity, and smoking status. This may limit the ability to draw conclusions about the relationship between OSTI scores and BMD T scores.

## Conclusions

In summary, OSTA is a simple and validated tool that can identify subjects at increased risk of osteoporosis. Its use could facilitate a more cost-effective use of BMD; by avoiding measurements in low-risk groups. Moreover, the management of osteoporosis is costly, so early detection and prevention will further lower costs. Osteoporosis risk is effectively identified by a cutoff score of -4. However, we used slightly different cutoffs to have higher sensitivity. Women, especially postmenopausal women, should use it to promote their health and prevent osteoporosis and its complications early.
